# SARS-CoV-2 spike ectodomain targets α7 nicotinic acetylcholine receptors

**DOI:** 10.1016/j.jbc.2023.104707

**Published:** 2023-04-13

**Authors:** Brittany C.V. O’Brien, Lahra Weber, Karsten Hueffer, Maegan M. Weltzin

**Affiliations:** 1 Department of Chemistry and Biochemistry, University of Alaska Fairbanks, Fairbanks, Alaska, USA; 2 Department of Veterinary Medicine, University of Alaska Fairbanks, Fairbanks, Alaska, USA

**Keywords:** allosteric modulator, cholinergic receptor, electrophysiology, glycoprotein, nicotinic acetylcholine receptor (nAChR), nicotine, peptide interaction, SARS-CoV-2

## Abstract

Virus entry into animal cells is initiated by attachment to target macromolecules located on host cells. The severe acute respiratory syndrome coronavirus 2 (SARS-CoV-2) trimeric spike glycoprotein targets host angiotensin converting enzyme 2 to gain cellular access. The SARS-CoV-2 glycoprotein contains a neurotoxin-like region that has sequence similarities to the rabies virus and the HIV glycoproteins, as well as to snake neurotoxins, which interact with nicotinic acetylcholine receptor (nAChR) subtypes *via* this region. Using a peptide of the neurotoxin-like region of SARS-CoV-2 (SARS-CoV-2 glycoprotein peptide [SCoV2P]), we identified that this area moderately inhibits α3β2, α3β4, and α4β2 subtypes, while potentiating and inhibiting α7 nAChRs. These nAChR subtypes are found in target tissues including the nose, lung, central nervous system, and immune cells. Importantly, SCoV2P potentiates and inhibits ACh-induced α7 nAChR responses by an allosteric mechanism, with nicotine enhancing these effects. Live-cell confocal microscopy was used to confirm that SCoV2P interacts with α7 nAChRs in transfected neuronal-like N2a and human embryonic kidney 293 cells. The SARS-CoV-2 ectodomain functionally potentiates and inhibits the α7 subtype with nanomolar potency. Our functional findings identify that the α7 nAChR is a target for the SARS-CoV-2 glycoprotein, providing a new aspect to our understanding of SARS-CoV-2 and host cell interactions, in addition to disease pathogenesis.

The novel strain severe acute respiratory syndrome coronavirus-2 (SARS-CoV-2) has caused a global pandemic of the potentially fatal coronavirus disease 2019 (COVID-19). SARS-CoV-2 has caused more than 650 million confirmed COVID-19 cases and more than 6.65 million deaths worldwide according to the Johns Hopkins Coronavirus Resource Center as of December 14, 2022. COVID-19 is a severe acute respiratory syndrome that initially infects respiratory epithelial cells by binding to the angiotensin converting enzyme 2 (ACE2) but other cellular targets have been proposed (1, 2, 3, 4, 5). SARS-CoV-2 infection results in severe inflammation and damage to peripheral organs (6, 7, 8). There is also increasing evidence that SARS-CoV-2 enters the central nervous system (CNS) and can have long-term effects including memory impairment and fatigue (9, 10).

Among hospitalized COVID-19 patients, a higher than expected smoking prevalence has been reported (11, 12, 13, 14, 15) although the reverse has also been proposed (16, 17). Whichever is the case, smokers who are hospitalized have an increased rate of symptom onset and experience worse outcomes (14, 18, 19). Smoking compromises the immune system and increases the risk for respiratory infections, chronic obstructive pulmonary diseases, lung cancer, and other conditions (20, 21, 22, 23, 24). Tobacco smoke increases ACE2 expression (15, 25), which is likely one reason why COVID-19 patients with chronic obstructive pulmonary disease or who are current smokers have increased ACE2 expression in bronchial epithelial cells in the respiratory tract compared with healthy subjects (15, 26, 27, 28, 29, 30, 31, 32). Nicotine, a main addictive component of tobacco products, targets nicotinic acetylcholine receptors (nAChRs) causing both activation and desensitization (33, 34, 35). Activation of the α7 nAChR subtype *via* nicotine causes an increase in ACE2 levels in epithelial cells, whereas gene silencing of the α7 nAChR appears to significantly dampen this response (30, 36). Further, the cholinergic anti-inflammatory pathway may play a role in smokers and COVID-19 disease severity as nicotine exposure attenuates the immune response *via* an α7 nAChR-mediated mechanism (37, 38, 39, 40).

nAChRs are pentameric ligand gated ion channels that are composed of different combinations of α (α2–10) and β (β2–4) subunits (41). α4β2 and α7 nAChR subtypes have been identified to be targets for the glycoproteins of the rabies virus and HIV, respectively (42, 43). The rabies virus, HIV, and SARS-CoV-2 glycoproteins all contain a conserved protein sequence similar to loop 2 of α-bungarotoxin (Table S1) (44). This neurotoxin-like region is critical for nAChR interactions with α-bungarotoxin, as well as the rabies virus and HIV glycoproteins (45, 46, 47, 48, 49). The SARS-CoV-2 glycoprotein is located on the surface of the virion, forming a homotrimer spike with two regions S1 and S2 which can interact with cellular targets to mediate host entry (50, 51). A structural model of the SARS-CoV-2 glycoprotein shows this neurotoxin-like region, located at the junction between the S1 and S2 segments, is highly exposed to solvent, free of shielding glycans, and accessible to host cellular protein targets (4, 52). Functional and binding studies have shown that the SARS-CoV-2 neurotoxin-like region functions either as a coagonist on α7 nAChRs and does not bind to the α7 nAChR orthosteric site, respectively (53, 54).

In this work, we set out to determine if the SARS-CoV-2 glycoprotein neurotoxin-like region targets nAChRs and if nicotine modifies this potential interaction. To do so, we used a SARS-CoV-2 glycoprotein peptide (SCoV2P) comprised of residues Y660-S689 of the spike ectodomain (YECDIPIGAGICASYQTQTNSPRRARSVAS) (Table S1) and the SARS-CoV-2 ectodomain (SCoV2ED) to investigate the functional effects on nAChRs using two-electrode voltage clamp (TEVC) electrophysiology. A neuronal-like cell culture model was used to verify the interaction of SCoV2P with α7 nAChRs, as well as confirm the lack of cytotoxicity for this peptide. The α7, α4β2, α3β4, and α3β2 nAChR subtypes were chosen as these are expressed in SARS-CoV-2 target tissues, including nose, lung, the CNS, and some immune cells (55, 56, 57, 58). We hypothesized that the ectodomain and SCoV2P would antagonize these nAChR subtypes, given the sequence similarities to α-neurotoxins and the rabies glycoprotein. Further, we anticipated that nicotine would enhance this potential inhibition effect.

Using SCoV2P, we defined that the neurotoxin-like region antagonizes nAChRs in a subtype selective manner, showing a high preference for the α7 subtype. Even more surprising, SCoV2P potentiates and inhibits acetylcholine (ACh)-induced α7 nAChR responses by a potential allosteric mechanism and nicotine enhances these effects. We further show that SCoV2P can reset desensitized α7 nAChRs by aiding receptor transition back to the resting closed state. Confocal imagining of α7 nAChR transfected cultured neuronal-like N2a and human embryonic kidney 293 (HEK293) cells confirmed that SCoV2P interacts with α7 nAChRs expressed on the cell surface. Importantly, we identified that the SCoV2ED potentiates and antagonizes the α7 nAChR subtype with nM potency. Our functional and *in vitro* cell culture findings confirm that the α7 nAChR is a target for the SARS-CoV-2 glycoprotein, providing a new aspect to our understanding of SARS-CoV-2 and host cell interactions.

## Results

### Heteromeric nAChR SCoV2P inhibition

**Figure 1:**
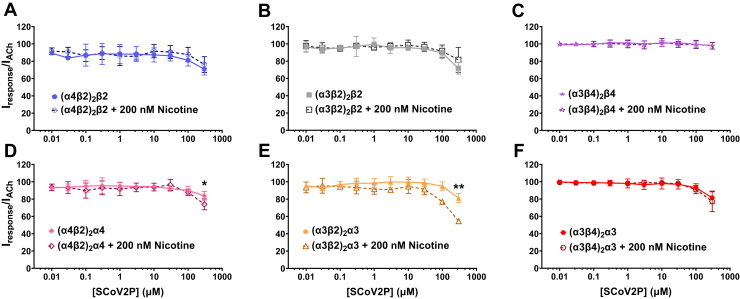
**SCoV2P minimally inhibits ACh-invoked heteromeric nAChR responses.** Data are grouped by isoform and normalized to ACh-induced responses without SCoV2P application. Significant changes are noted with ∗ and were determined for 300 μM SCoV2P difference between nicotine naïve and 200 nM nicotine preincubation responses (Welch’s two-tailed *t* test: (α4β2)_2_α4 *t* (7.2) = 2.57, ∗*p* = 0.036; (α4β2)_2_α3 *t* (6.8) = 4.46, ∗∗*p* = 0.0032). *A*–*C*, high agonist sensitivity nAChRs were minimally inhibited with SCoV2P and nicotine treatment had no effect. *D*–*F*, low agonist sensitivity nAChRs experienced an increase in SCoV2P-induced inhibition with nicotine presence. Points are the mean ± SD (N = 3, n = 3–5). ACh, acetylcholine; nAChR, nicotinic acetylcholine receptor; SCoV2P, SARS-CoV-2 glycoprotein peptide.

Modeling data has predicted that the SCoV2ED neurotoxin-like region interacts with α4β2 and α7 nAChRs (4). We performed SCoV2P concentration-response curves using heteromeric α4β2, α3β2, and α3β4 nAChRs and the α7 homomer (presented in the following sections) to test if SCoV2P functionally interacts with these nAChRs. Heteromeric nAChRs express in distinct stoichiometries. High agonist sensitivity isoforms form with two αβ pairs and one additional β subunit. In addition to the two αβ pairs, the low agonist sensitivity isoforms have an accessory α subunit. Preapplication of SCoV2P in absence of ACh did not activate any of the tested nAChRs (Fig. S1*A*). SCoV2P applied to non- circular RNA (cRNA) injected oocytes did not induce a response in the absence or presence of ACh (Fig. S1*B*). Application up to 100 μM of SCoV2P caused no change in ACh-induced responses, which was followed by a minimal antagonistic effect with 300 μM SCoV2P applied to heteromeric nAChRs in absence of nicotine (Fig. 1). For α4β2 and α3β2 nAChRs, the low agonist sensitivity isoforms experienced less inhibition than the high agonist sensitivity isoforms. The (α3β4)_2_β4 isoform ACh currents were completely unmodified by SCoV2P application (Fig. 1*C*).

To determine if nicotine altered SCoV2P inhibition of α4β2, α3β2, and α3β4 nAChRs, nAChR-expressing oocytes were preincubated with 200 nM nicotine, a subactivating concentration found in the blood of moderate smokers (59, 60, 61), for 80 to 110 min. In the continual presence of nicotine, nAChRs were exposed to 30 s of increasing SCoV2P concentrations and then acutely stimulated with ACh for 1 s (Fig. 1). Nicotine pretreatment had no effect on SCoV2P inhibition of ACh-mediated currents for all high agonist sensitivity isoforms (Fig. 1, *A*–*C*). Low agonist sensitivity isoforms (α4β2)_2_α4 and (α3β2)_2_α3 were significantly inhibited by SCoV2P with nicotine preincubation (∗*p* = 0.036 and ∗∗*p* = 0.0032, respectively) (Fig. 1, *D*–*F*). These results show that the α(+)/α(−) interface may be important for SCoV2P interactions with nAChRs.

### SCoV2P modulation of homomeric α7 nAChRs

**Figure 2:**
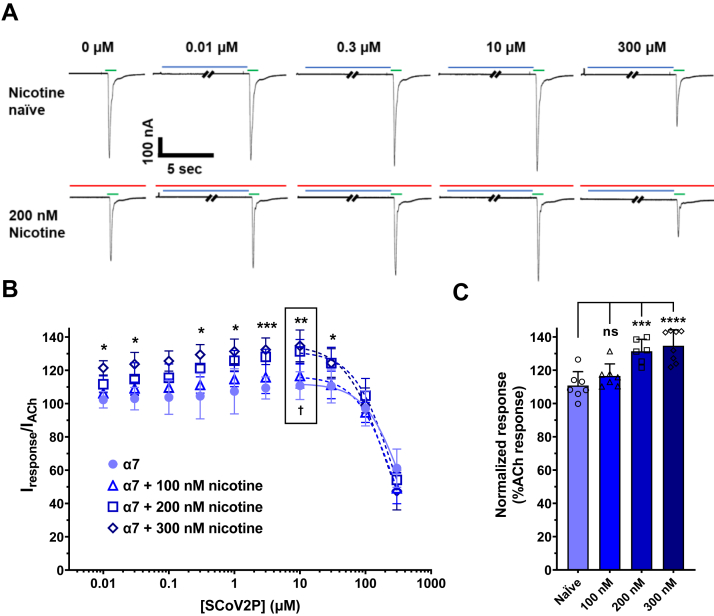
**SCoV2P modulation of homomeric α7 nAChRs.***A*, current recordings generated using cRNA injected *Xenopus laevis* oocytes displaying alterations in ACh-induced currents after preapplication of increasing SCoV2P concentrations (0.01–300 μM). Thirty seconds SCoV2P preapplications are shown in *blue*, while 1 s ACh stimulations are displayed in *green* (coloring scheme is applicable to all displayed recordings). *B*, normalized SCoV2P-induced alterations in ACh-induced responses as a percentage of control response. SCoV2P significantly potentiates (Two-tailed one sample *t* test *t* (4) = 3.41, ^†^*p* = 0.0271) and inhibits ACh-induced currents. Nicotine 200 and 300 nM pretreatments augment the SCoV2P effect. Significant changes are noted with ∗ to indicate differences between nicotine naïve and 200 nM nicotine treatment group (N = 3–5, n = 7–9) (Welch’s two-tailed *t* test *t* (4.3–7.9) = 2.28–5.77, ∗*p* < 0.05, ∗∗*p* = 0.0014, ∗∗∗*p* = 0.0005). The box identifies maximal potentiation induced using 10 μM SCoV2P. All points are the mean±SD. *C*, ten μM SCoV2P significantly potentiates ACh currents, which are further enhanced with increasing nicotine concentrations (One-Way ANOVA with Tukey’s posthoc analysis F (3, 24) = 14.00, ∗∗∗*p* = 0.0005, ∗∗∗∗*p* < 0.0001). ACh, acetylcholine; nAChR, nicotinic acetylcholine receptor; SCoV2P, SARS-CoV-2 glycoprotein peptide.

To uncover if SCoV2P alters homomeric α7 nAChR function, we performed SCoV2P concentration-response experiments using α7 nAChRs naïve or preincubated with 100, 200, or 300 nM nicotine for 80 to 110 min (Fig. 2). For peptide concentration-response curves, oocytes were exposed to 30 s of SCoV2P followed by 1 s application of ACh at the concentration that induced 90 percent of the maximal response (EC_90_), which we determined to be 1300 μM. α7 nAChR response traces display that for the naïve and nicotine pretreatment groups, increasing concentrations of SCoV2P potentiate ACh peak currents (0.01–10 μM) before inhibiting ACh responses (30–300 μM) (Fig. 2*A*, quantified in 2*B*). In the absence of nicotine, the SCoV2P potentiation phase was slight, with a maximum value of 111 ± 8% (^†^*p* = 0.0271) (Fig. 2*B* and Table S2). The inhibitory component of SCoV2P reduced the α7 nAChR ACh-induced response by 39% with a potency of 337 μM (95% confidence interval [CI] [lower limit, upper limit] [273, 460]) (Table S2). These results identify that the SCoV2ED neurotoxin-like region modulates α7 nAChR function. The increase in SCoV2P-mediated inhibition of ACh-induced currents of the α7 nAChR over the heteromeric α4β2, α3β2, and α3β4 nAChRs provides further support that the α (+)/α (−) interfaces are critical for SCoV2P actions.

Pretreating α7 nAChRs with 100, 200, or 300 nM nicotine produced SCoV2P concentration-response profiles that identified differences to the nicotine naïve α7 nAChR profile (Fig. 2*B* and Table S2). Preexposing with either 200 or 300 nM nicotine enhanced the potentiated ACh-evoked currents to 131 ± 7% (∗∗*p* = 0.0016) or 135 ± 10% (∗∗*p* = 0.0015), respectively compared to the nicotine naïve currents (Fig. 2*B* and Table S2). Nicotine pretreatment increased inhibition phase potency from 337 μM [CI 273, 460]) (nicotine naïve) to 197 μM [CI 166, 231] (300 nM pretreatment) (∗*p* = 0.0246) (Table S2). These findings demonstrate that nicotine exposure enhances SCoV2P potentiation (Fig. 2, *B* and *C*) and inhibition of α7 nAChR ACh-induced currents (Fig. 2*B*), in a concentration-dependent manner. The ability of nicotine to enhance SCoV2P potentiation could be caused by nicotine pretreatment upregulating α7 nAChRs, augmenting the total receptor pool, and thus enhancing SCoV2P potentiation. Alternatively, SCoV2P is an allosteric modulator that resensitizes nicotine desensitized α7 nAChRs. We explored both possibilities.

### Nicotine-driven receptor dynamics

**Figure 3:**
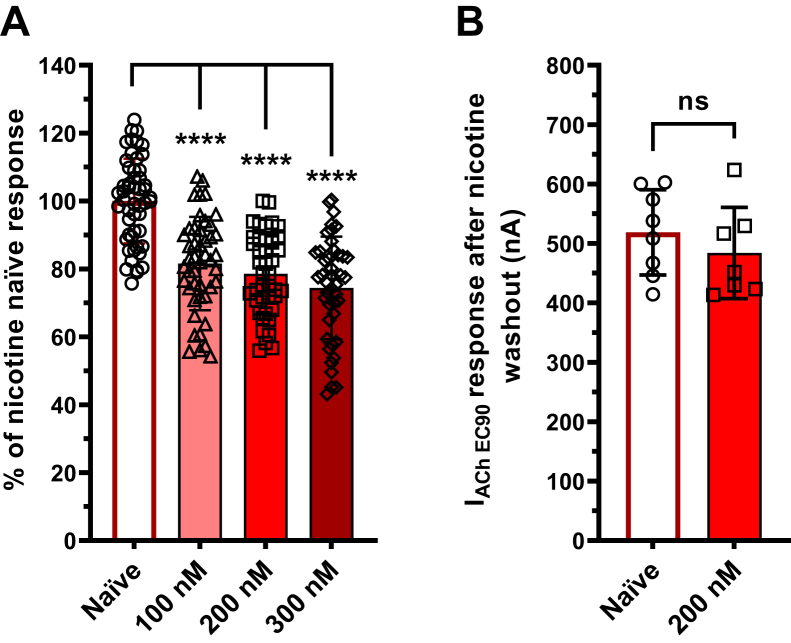
**Nicotine driven α7 nAChR desensitization.***A*, nicotine desensitized α7 nAChRs in a concentration-dependent manner (*right*) (N = 4, n = 42–52) (One-Way ANOVA with Dunnett's posthoc analysis, F (3,12) = 32.09, ∗∗∗∗*p* < 0.0001). Data was collected at the second ACh application after full-agonist response has been demonstrated (96). *B*, upon recovery, nicotine pretreated α7 nAChRs showed no increase in macroscopic ACh responses (*left*) (N = 3, n = 7–9) (Welch’s two-tailed *t* test *t* (4) = 0.33, ^ns^P = 0.757). All points are the mean ± SD. ACh, acetylcholine; nAChR, nicotinic acetylcholine receptor.

It is well documented that prolonged nicotine exposure can desensitize α7 nAChRs (62). To quantify the relative amount of α7 nAChRs desensitized by clinically relevant concentrations of nicotine, oocytes were pretreated with 100 nM, 200 nM, or 300 nM nicotine for 80 to 110 min and ACh-evoked currents were measured (Fig. 3*A*). When compared with nicotine naïve oocytes, pretreatment did desensitize a significant portion of α7 nAChRs (∗∗∗∗*p* < 0.0001). Pretreatment of 100 nM nicotine desensitized 18 ± 14% of α7 nAChRs, while 200 nM nicotine desensitized 21 ± 12% and 300 nM nicotine pretreatment desensitized maximally 26 ± 15% of the total activatable pool.

It is also known that nicotine exposure can increase nAChR expression on the cell surface (63, 64, 65). Although unlikely, if our nicotine pretreatment chaperoned α7 nAChRs to the cell surface, then upon recovery from desensitization, the total ACh peak current would be augmented compared to the nicotine naïve group. To test this, α7 nAChRs were preincubated with 200 nM nicotine and tested as described above. ACh peak currents were recorded every 4 min until the α7 nAChRs recovered from desensitization (∼24 min) (Fig. S2). We observed no change in total ACh peak current response between the naïve and the recovered 200 nM nicotine treatment groups (Fig. 3*B*). These findings demonstrate that nicotine preincubation did not alter the total functional receptor pool, thus the SCoV2P potentiation we observed in Figure 2, *B* and *C* is likely due to allosteric actions.

### α7 nAChR allosteric modulation by SCoV2P

**Figure 4:**
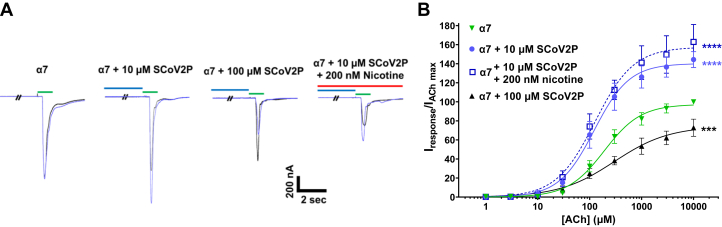
**SCoV2P allosteric modulation of α7 nAChRs.***A*, raw recordings showing ACh maximal (10 mM) currents in the absence (*black*) and presence (*blue*) of 10 or 100 μM SCoV2P and preincubated with 200 nM nicotine (10 μM SCoV2P). Each set of traces were collected from unique oocytes. *B*, ten μM SCoV2P enhances ACh potency, in addition to enhancing ACh-induced responses significantly. A higher SCoV2P concentration (100 μM) reduced ACh potency and efficacy (One-Way ANOVA with Tukey’s posthoc analysis F (3,13) = 144.8, ∗∗∗*p* = 0.0003, ∗∗∗∗*p* < 0.0001). All points are the mean±SD. Data values are reported in Tables S2 and S3. ACh, acetylcholine; nAChR, nicotinic acetylcholine receptor; SCoV2P, SARS-CoV-2 glycoprotein peptide.

To determine if SCoV2P is an allosteric modulator of α7 nAChRs, ACh concentration-response curves were generated in the presence of either the maximum potentiating concentration of SCoV2P (10 μM) or a high inhibiting concentration (100 μM) and compared to SCoV2P naïve ACh responses (Fig. 4*A*). With preapplication of 10 μM SCoV2P, the ACh potency was significantly enhanced from 192 μM [CI 174, 212] to 119 μM [CI 104, 138]), as noted by the leftward shift of the response curve (∗∗*p* = 0.0060) (Fig. 4*B* and Table S3). The 200 nM nicotine pretreatment group also displayed enhanced SCoV2P potency (121 μM [CI 103, 150])) when compared with the ACh-only control (nicotine naïve, no SCoV2P) group (∗∗*p* = 0.0014) (Fig. 4*B* and Table S3). Preapplication of 10 μM SCoV2P increased ACh efficacy, as noted by the upward shift of the response curve, from the α7 nAChR control (99.9 ± 0.3%) to 144 ± 8% (∗∗∗∗*p* < 0.0001). The 200 nM nicotine pretreatment further enhanced the SCoV2P potentiation to 163 ± 18% (∗∗∗∗*p* < 0.0001) (Fig. 4*B* and Table S3).

In addition to SCoV2P potentiating α7 nAChR ACh-mediate responses, higher concentrations of SCoV2P inhibited ACh responses. Using 100 μM SCoV2P, ACh potency (287 μM [CI 195, 481]) was reduced when compared to untreated α7 nAChRs (Fig. 4*B* and Table S3). ACh efficacy was also reduced reaching a maximum of 73 ± 9% when compared to α7 nAChR ACh only responses (∗∗∗*p* = 0.0003). Due to SCoV2P preapplication causing shifts in both ACh potency and efficacy, our data demonstrate that SCoV2P is an allosteric modulator of α7 nAChRs.

### SCoV2P mechanism of action

**Figure 5:**
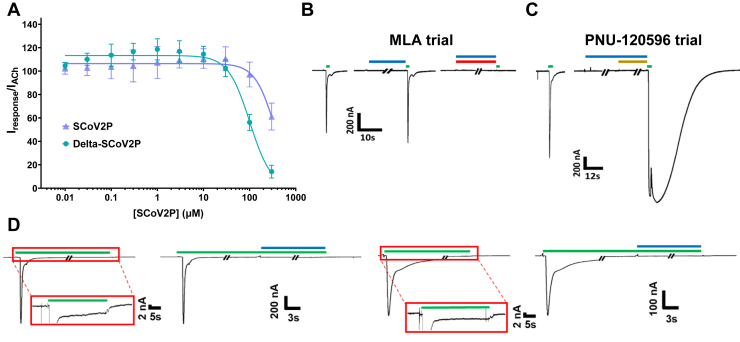
**α7 nAChR current responses to pharmacological ligands reveals SCoV2P mechanisms of action.***A*, normalized delta SCoV2P-induced alterations in ACh-induced responses as a percentage of control response. Previously presented SCoV2P data is included to facilitate comparison. The single point mutation found in the neurotoxin-like region caused a significant change in inhibition potency (One-Way ANOVA with Tukey’s posthoc analysis F (4, 14) = 10.04, ∗∗∗*p* = 0.0002). *B*, MLA (*red*) blocks SCoV2P (*blue*) and ACh (*green*)-induced currents. *C*, SCoV2P coapplied with PNU-120596 (*gold*) does not induce a current nor does SCoV2P block PNU-120596 modulation of ACh-mediate currents. *D*, SCoV2P is not capable of directly reactivating desensitized receptors but instead facilitate receptors to recover from being desensitized (*i.e.*, return to the closed state). Experiments were conducted at ACh EC_100_ (*top*) and at EC_20_ (*bottom*) concentrations. The red box enlargement is showing that receptors are in steady state after a 30 s application of ACh. All experiments were performed on at least two individual animals and at least three oocytes. ACh, acetylcholine; MLA, methyllycaconitine; nAChR, nicotinic acetylcholine receptor; SCoV2P, SARS-CoV-2 glycoprotein peptide.

Being a relatively small peptide, SCoV2P could interact with α7 nAChRs in many ways, including opportunistically. To demonstrate that SCoV2P interactions are mediated by specific residues, we tested the delta variant mutation (P681R). This single point mutation enhanced inhibition phase potency from 337 μM [CI 273, 490] to 100 μM [CI 91,111] without significantly changing potentiation in comparison to SCoV2P (Fig. 5*A*). These data demonstrate that residue 681 can mediate specific interactions with α7 nAChRs to alter, and in the case of P681R, enhance potency.

To further ensure that the effects observed with SCoV2P were α7 nAChR specific, we blocked SCoV2P potentiation with the competitive antagonist methyllycaconitine (MLA) (66). MLA coapplied with SCoV2P fully prevented α7 nAChRs response to ACh, verifying that the SCoV2P effects are mediated by α7 nAChRs (Fig. 5*B*). To evaluate if SCoV2P is a silent agonist as recently proposed by Chrestia *et al*. (2022), we explored if SCoV2P could induce receptor activation in the presence of the positive allosteric modulator PNU-120596 (53). To test this, we preapplied 3 μM SCoV2P followed by coapplication of 3 μM SCoV2P and 3 μM PNU-120596. We lastly stimulated the α7 nAChRs with ACh to demonstrate that the receptors were functional (Fig. 5*C*). Following this protocol, we observed no current responses when SCoV2P and PNU-120596 were both present. With application of ACh, the response was massively potentiated demonstrating that the α7 nAChRs were functional. Because SCoV2P did not alter or block the effects of PNU-120596, nor did the combination of these two molecules activate the receptors in the absence of ACh, it is likely that SCoV2P does not share a common binding pocket with ACh or PNU-120596 on the α7 nAChR.

As SCoV2P facilitates α7 nAChR recovery from nicotine desensitization (Figs. 2 and 3), we wanted to determine if SCoV2P can directly reactivate desensitized receptors. We applied fully saturating and desensitizing concentrations of ACh (30 mM) for approximately 30 s until the steady state was reached (Fig. 5*D* top). We then coapplied ACh with potentiating concentrations of SCoV2P and found that SCoV2P had no effect on the steady-state response (Fig. 5*D* left). The same experiment was replicated using a lower concentration of ACh (EC_20_ 60 μM) and again we observed no change in the steady state response (Fig. 5*D* right). These experimental results demonstrate that for SCoV2P to modulate α7 nAChRs, it must first be able to interact with the receptors prior to application of ACh (Fig. 5*D* right). Further, when SCoV2P is applied alone to nicotine desensitized receptors, no activation occurs (Fig. S3). Based on the inability of SCoV2P to directly reactivate desensitized receptors (*i.e.*, transitioning from the receptor desensitized state directly to the open state) (Fig. S3) it is likely that SCoV2P is acting allosterically *via* a mechanism that can transition receptors from the desensitized to the resting state.

To ensure that the effects of SCoV2P on α7 nAChRs is sequence specific, and not a result of opportunistic effects, we used a peptide of the Pasteur lab strain rabies viral glycoprotein (RVG-P) containing the neurotoxin-like motif. This peptide (RVG-P) is the same length as SCoV2P (30-mer), similar amino acid composition and has high sequence homology in the neurotoxin domain (Table S1). Functionally, the RVG-P, unlike SCoV2P, did not potentiate α7 nAChR ACh-mediated responses at low concentrations (Fig. S5). Instead, RVG-P antagonized ACh-induced currents and is approximately 30 times more potent that SCoV2P, with an IC_50_ of 10 μM [9, 11] than 337 μM [273, 460] for SCoV2P. These large differences in both potentiation and inhibition verify that the SCoV2P’s effects on the α7 nAChR are unique.

### SCoV2P region Y674-S689 allosterically modulates α7 nAChRs

**Figure 6:**
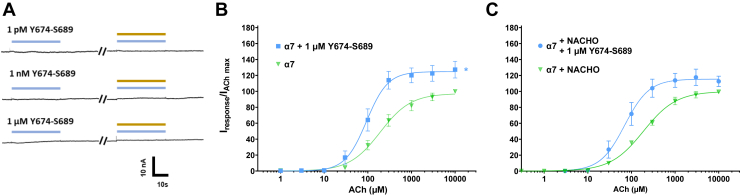
**Y674-S689 positively modulates α7 nAChRs expressed in absence and with the chaperone protein NACHO.***A*, raw recordings showing currents in presence of 1 μM Y674-S689 (*light blue*) and 10 μM PNU-120596 (*gold*) show that no α7 nAChR activation occurred. Each set of traces were collected from unique oocytes (N = 3, n = 3–5). *B*, one μM Y674-S689 significantly enhanced ACh potency (One-Way ANOVA with Tukey’s posthoc analysis F (3, 9) = 69.12, ∗∗∗∗*p* < 0.0001) and efficacy (One-Way ANOVA with Tukey’s posthoc analysis F (3, 9) = 6.682, ∗*p* = 0.0163). *C*, α7 nAChRs co-expressed with NACHO showed similar and 1 μM Y674-S689 responses as α7 nAChRs expressed alone, with 1 μM Y674-S689 significantly enhancing ACh potency (One-Way ANOVA with Tukey’s posthoc analysis F (3, 9) = 69.12, ∗∗∗∗*p* < 0.0001). All points are the mean ± SD. Data values are reported in Tables S4. ACh, acetylcholine; nAChR, nicotinic acetylcholine receptor.

While the present article was in preparation, Chrestia *et al*. (2022) published work demonstrating that a shorter version of SCoV2P (residues Y674-R685) was an α7 silent agonist, as this peptide could cause α7 nAChR activation when PNU-120596 was coapplied in absence of agonist (53). To follow Chrestia *et al*. (2022) studies, we co-applied a shortened SCoV2P peptide (Y674-S689) with 10 μM PNU-120596 at three different peptide concentrations (1 PM, 1 nM, and 1 μM). In all drug conditions, we saw no α7 nAChR activation without application of agonist (Fig. 6*A*).

To determine if Y674-S689 could function as an allosteric modulator, we performed ACh concentration response profiles in presence of 1 μM Y674-S689 (Fig. 6*B*). We observed significant potentiated shifts in both potency 93 μM [85, 102] (∗∗∗∗*p* < 0.0001) and efficacy (127 ± 11) (∗*p* = 0.0378) as compared to peptide naïve α7 nAChRs (Fig. 6*B* and Table S4). One difference between our work and Chrestia *et al*. (2022) is the use of the chaperone protein NACHO (53). When we co-expressed α7 nAChR with NACHO, we observed no difference in ACh potency or maximal currents compared to α7 nAChRs expressed without NACHO (Fig. 6*C*). This was not unexpected as *Xenopus laevis* oocytes contain endogenous chaperone proteins, allowing for α7 nAChRs to readily express. Using α7 nAChRs co-expressed with NACHO, the change in ACh potency with application of 1 μM Y674-S689 was the same as α7 nAChR expressed without NACHO (Fig. 6*C* and Table S4).

### Visualization of SCoV2P interacting with α7 nAChR-positive neuronal-like cells

**Figure 7:**
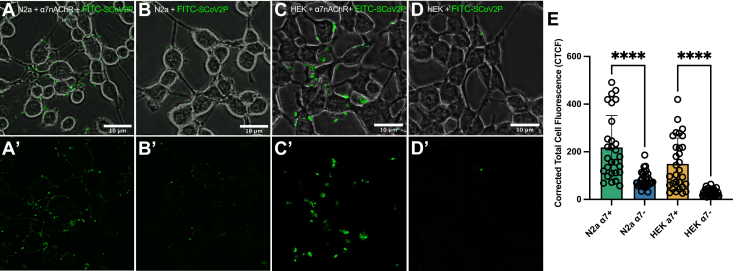
**SCoV2P preferentially interacts with cells expressing α7 nAChRs.** Live-cell confocal images of 50 μM FITC-SCoV2P treated (*A*) α7 nAChR-transfected N2a, (*B*) nontransfected N2a, (*C*) α7 nAChR-transfected HEK293, and (*D*) α7-null HEK293 cells. (*A*′–*D*′) Same as (*A*–*D*) without displaying the phase channel. *E*, CTCF of 24 h incubation of FITC-SCoV2P with α7 nAChR-transfected N2a and HEK293 cells, as well as nontransfected N2a and HEK293 cells (number of analyzed cells (n) = 30, student’s unpaired *t* test t = 5.350, df = 56 (N2a); t = 5.983, df = 58 (HEK293), ∗∗∗∗*p* < 0.0001). All points are mean ± SD. CTCF, corrected total cell fluorescence; HEK293, human embryonic kidney 293; nAChR, nicotinic acetylcholine receptor; SCoV2P, SARS-CoV-2 glycoprotein peptide.

To further demonstrate SCoV2P interacts with α7 nAChRs in a cellular context, we applied FITC N-terminally labeled SCoV2P to N2a and HEK293 cells. N2a cells were chosen because they are neuronal-like and commonly used to study nAChRs, but do endogenously express low levels of nAChRs (67, 68), while HEK293 cells are nAChR-negative. N2a and HEK293 cells transiently transfected with α7 nAChR and NACHO were abundantly labeled with FITC-SCoV2P (Fig. 7, *A* and *C*). Non-transfected N2a cells show slight fluorescence labeling but this is not unexpected as N2a cells endogenously express nAChRs (Fig. 7*B*). HEK293 cells, which do not endogenously express nAChRs, display no fluorescence (Fig. 7*D*). Analysis of corrected total cell fluorescence (CTCF) showed a significant increase in fluorescence for FITC-SCoV2P treated α7 nAChR transfected N2a and HEK293 cells compared to non-transfected cells (Fig. 7*E*). We evaluated SCoV2P cytotoxicity and found that SCoV2P did not induce cell death at any tested concentration (1–100 μM) (Fig. S6). As dimethyl sulfoxide (DMSO) is cytotoxic, a 15% DMSO control was used to demonstrate detection of dead cells in this assay.

**Figure 8:**
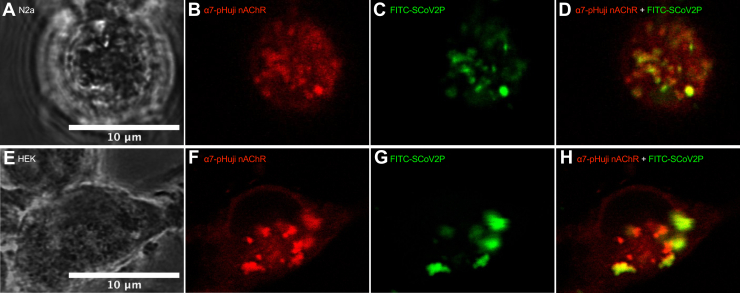
**FITC-SCoV2P interacts with α7-pHuji nAChRs expressed on the plasma membrane.***A*, phase channel, (*B*) FITC-channel, (*C*) pHuji-channel, and (*D*) merged live-cell confocal images of an α7-pHuji nAChR-transfected N2a cell exposed to 50 μM FITC-SCoV2P for 24 h prior to imaging.(*E*–*H*, same as (*A*–*D*) for an HEK293 cell. Merged images demonstrate colocalization of FITC-SCoV2P and α7-pHuji nAChRs. HEK293, human embryonic kidney 293; nAChR, nicotinic acetylcholine receptor; SCoV2P, SARS-CoV-2 glycoprotein peptide.

To further validate that SCoV2P interacts with α7 nAChRs expressed on the cell surface, we used α7 nAChRs that have the fluorescent probe pHuji attached to the α7 subunit Cterminal (α7-pHuji) and transfected N2a and HEK293 cells as above. Application of 50 μM FITC-SCoV2P shows colocalization with α7-pHuji nAChRs and further confirms our functional data demonstrating that SCoV2P interacts with α7 nAChRs (Fig. 8).

### SCoV2ED potently modulates α7 nAChRs

**Figure 9:**
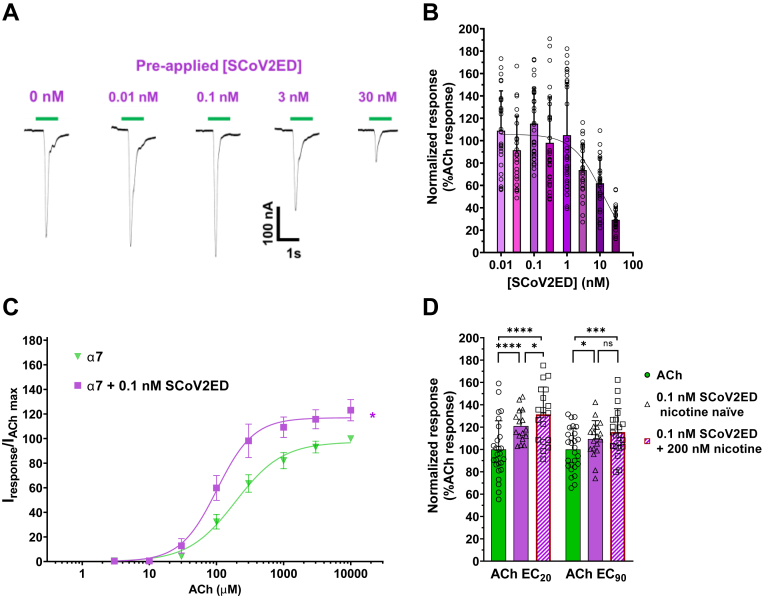
**SCoV2ED potentiates and inhibits ACh α7 nAChRs with nanomolar potency.***A*, α7 nAChR ACh-evoked currents were measured by TEVC using cRNA injected *Xenopus laevis* oocytes. SCoV2ED (0.01–30 nM) was applied for 5 min prior to ACh stimulation. Green drug application bars indicate the time 1300 μM ACh was applied. *B*, increasing concentrations of SCoV2ED inhibited ACh-induced responses. Data were normalized to the control response (40% PBS). Data are the mean±SD (N = 5, n = 25–44). Nonlinear regression curve fit to SCoV2ED data with an IC_50_ of 11 ± 6 nM. *C*, 0.1 nM SCoV2ED enhances ACh potency from 192 μM [CI 174, 212] to 102 μM [CI 93, 111] (student’s unpaired *t* test, t = 4.625, df = 6, ∗∗*p* = 0.0036). ACh maximal responses were also significantly enhanced (123 ± 9%) (student’s unpaired *t* test t = 3.207, df = 5, ∗*p* = 0.0238) when compared to α7 nAChRs that were not exposed to SCoV2ED (N = 3–5, n = 9–14). *D*, at the ACh EC_20_ and EC_90_, 0.1 nM SCoV2ED (*solid purple bar*) significantly potentiated the ACh-mediated response (*solid green bar*). With 200 nM nicotine preincubation (*hashed purple bar*), 0.1 nM SCoV2ED significantly potentiated the ACh-induced control response (40% PBS) and in the case of the EC_20_ data greater than the 0.1 nM SCoV2ED enhanced currents (N = 3, n = 16–21) (One-way ANOVA with Tukey’s multiple comparison test F(5,12) = 49.85, ^ns^P = 0.2715, ∗*p* < 0.05, ∗∗∗*p* = 0.0007, ∗∗∗∗*p* < 0.0001). Data are the mean ± SD. ACh, acetylcholine; nAChR, nicotinic acetylcholine receptor; SCoV2ED, SARS-CoV-2 ectodomain; TEVC, two-electrode voltage clamp.

Many of the commercially available SARS-CoV-2 glycoprotein ectodomains have mutations within the neurotoxin-like region, including the ^682^RRAR^685^ furin cleavage site, where it is hypothesized, and we have demonstrated (Fig. 5*A*) nAChR interactions occur (1, 4). To ensure that the potential neurotoxin-like region interaction site was intact, we obtained an unmutated, furin-cleavable SCoV2ED and performed modified concentration-response experiments on the lung and CNS abundant α7 subtype (Fig. 9). To provide sufficient time for the ectodomain to interact with surface α7 nAChRs, each oocyte was incubated with a single SCoV2ED concentration (0.01–30 nM) for 5 min, followed by a 1 s ACh EC_90_ stimulation (Fig. 9*A*). The resulting ACh-induced responses were both potentiated and inhibited at lower SCoV2ED concentrations (0.01–1 nM). With application of higher concentrations (3–30 nM), ACh responses were antagonized with a SCoV2ED potency of 12 nM [CI 8, 18] (Fig. 9*B*).

To further explore the potentiation seen at 0.1 nM SCoV2ED, we performed ACh concentration-response curves as described previously by preapplying SCoV2ED for 60 s before applying 1 s of increasing concentrations of ACh (1 μM – 10 mM). Responses were normalized to an application of 10 mM ACh without SCoV2ED preapplication (Fig. 9*C*). ACh potency was significantly enhanced from 192 μM [CI 174, 212] to 102 μM [CI 93, 111] with 0.1 nM SCoV2ED (student’s unpaired *t* test, t = 4.625, df = 6, ∗∗*p* = 0.0036). ACh maximal responses were also significantly enhanced (123 ± 9%) (student’s unpaired *t* test t = 3.207, df = 5, ∗*p* = 0.0238) when compared to α7 nAChRs that were not exposed to SCoV2ED. Our findings generated with SCoV2ED are consistent with those obtained with SCoV2P. Potency differences are likely due to increased structural restraints of SCoV2ED compared to SCoV2P.

To examine how nicotine affects the potentiation seen at 0.1 nM SCoV2ED, a similar set of experiments was performed as above (Fig. 2*B*), with nicotine naïve and 200 nM nicotine preexposure groups. Experiments were performed at both the ACh EC_20_ and EC_90_. With application of 0.1 nM SCoV2ED, the ACh EC_20_ (121 ± 14%, ∗∗∗∗*p* < 0.0001), and EC_90_ (109 ± 16%, ∗*p* = 0.0101) responses were significantly potentiated compared to the ACh control responses (Fig. 9*D*). With 200 nM nicotine preexposure, 0.1 nM SCoV2ED further potentiated ACh-mediated currents at both the EC_20_ (131 ± 25%, ∗∗∗∗*p* < 0.0001) and EC_90_ (116 ± 21%, ∗∗∗*p* = 0.0007). SCoV2ED potentiated nicotine naïve and nicotine treated ACh EC_20_ responses more than the ACh EC_90_ responses (student’s unpaired *t* test, nicotine naïve t = 2.181, df = 32, ∗*p* = 0.0366, and nicotine pretreatment t = 2.208, df = 38, ∗*p* = 0.0333) (Fig. 9*D*). For physiological context, the SARS-CoV-2 spike glycoprotein binds to ACE2 with a dissociation constant of ∼15 nM (51). Our data newly identifies the ectodomain of SARS-CoV-2 spike glycoprotein to have a very high α7 nAChR functional potency.

## Discussion

Approximately one-third of COVID-19 patients experience neurological manifestations including delirium, psychiatric disorders, brain inflammation, hyposmia, cognitive deficits, stroke, and nerve damage (69, 70, 71). nAChRs are widely distributed across the CNS and are involved in neuroinflammation, psychiatric disorders, and other conditions (72, 73). Identifying host cellular targets of the SARS-CoV-2 spike glycoprotein will aid the development of treatment strategies to help those infected with COVID-19. We have demonstrated a functional interaction of the SARS-CoV-2 spike glycoprotein with nAChRs. Using a peptide of the SCoV2ED neurotoxin-like region, we identified that this region potentiates and inhibits α7 nAChRs in a concentration-dependent manner by an allosteric mechanism, while inhibiting α4β2, α3β4, and α3β2 subtypes minimally. We further demonstrate that SCoV2ED potentiates and inhibits α7 nAChR ACh-mediated currents with nanomolar potency. Nicotine treatments further enhanced the actions of both SCoV2P and SCoV2ED. Understanding how SCoV2P and SCoV2ED affect different nAChR subtypes and associated isoforms is likely relevant to understanding COVID-19 pathophysiology.

Prediction that the neurotoxin-like region of the SARS-CoV-2 glycoprotein interacts with nAChRs was initially suggested by Changeux (44). Modeling data has since predicted that this region interacts with α7 and α4β2 nAChRs (4). Interestingly, this *in silico* study predicted that the neurotoxin-like glycoprotein region interacts with high positional and conformational variability with the α7 and α4β2 nAChR models. In the α4β2 model, the peptide was not able to bind deeply into the proposed orthosteric-binding site, keeping the C loops of the receptor in the open conformation. The ability of the C loop to close over the binding pocket is an important step in achieving gating (74, 75, 76). Agonists are proposed to stabilize a compact C loop conformation, whereas antagonists prevent C loop closure. In the α7 nAChR model, the peptide showed multiple modes of C loop movement, including an open conformation and a semiclosed structure as the peptide moved deeper into the binding pocket (4). These modeling predictions are consistent with our functional data. The heteromeric α4β2, α3β4, and α3β2 subtypes were antagonized slightly with high concentrations of SCoV2P (Fig. 1). However, our α7 nAChR data was more complex, showing that the SCoV2P potentiates ACh-induced currents at low peptide concentrations, followed by inhibition at higher concentrations (Fig. 2). A point of difference is that our functional data show that SCoV2P binds to nAChRs *via* an allosteric mechanism, rather than at the orthosteric-binding site (Fig. 4). However, it seems possible that since the modeled peptide showed high positional and conformational variability, SCoV2P may interact with nAChRs in multiple orientations and/or sites and thus cause different functional outcomes.

**Figure 10:**
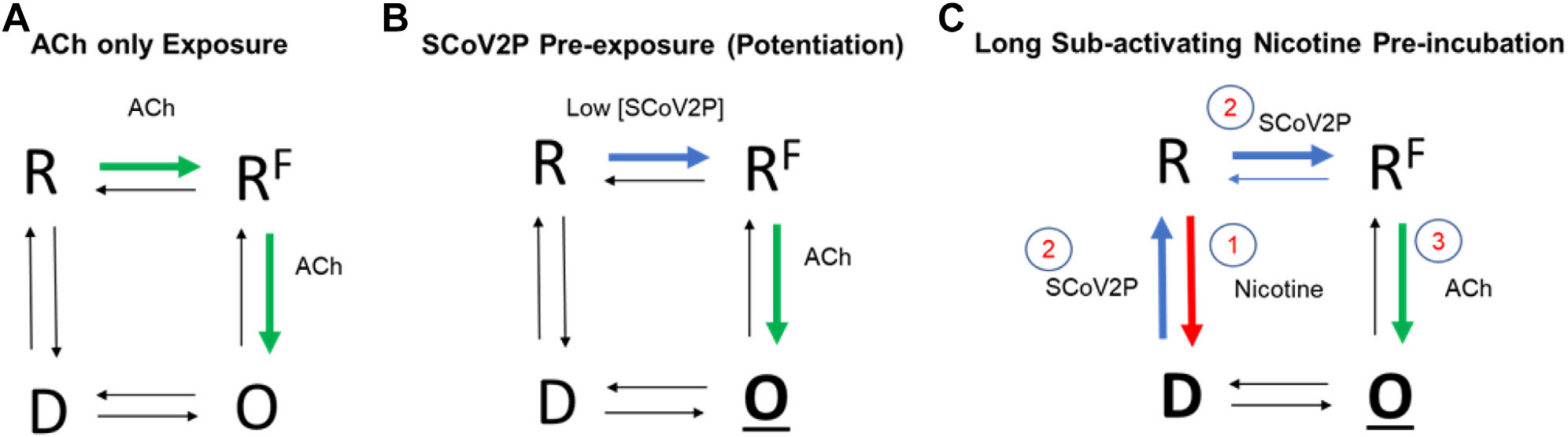
**Simplified SCoV2P mechanism of action on α7 nAChRs.***A*, application of ACh can transition α7 nAChRs from the closed, resting (R) state to an open (O), ion conducting state before the receptor transitions to the agonist-insensitive desensitized (D) state. It is thought that α7 nAChRs transition through an intermediate, nonconducting state (*e.g.*, the flip [R^F^] state) prior to the open conformation (38). The shown schematic is simplified and does not show the multiple ACh-binding sites and associated transition states. *B*, SCoV2P is an allosteric modulator capable of modifying α7 nAChR ACh potency and efficacy in a concentration-dependent manner. At low peptide concentrations, our data support the conclusion that SCoV2P can transition α7 nAChRs from the resting state to an intermediate state. The structural changes induced by SCoV2P to prime α7 nAChRs for rapid activation may allow conformation changes elicited by an agonist to be additionally stable or occur more rapidly, ultimately resulting in potentiated (O) ACh-mediated responses. *C*, SCoV2P is also able to transition α7 nAChRs from the nicotine-driven desensitized state back to the closed state. ACh, acetylcholine; SCoV2P, SARS-CoV-2 glycoprotein peptide; nAChR, nicotinic acetylcholine receptor.

Our data show that SCoV2P is an allosteric modulator of α7 nAChRs. With potentiating concentrations of SCoV2P preapplied prior to ACh, we observed a significant enhancement in ACh potency and efficacy (Fig. 4). These data suggest that SCoV2P can bind to α7 nAChRs and prime them for agonist activation (Fig. 10*B*). Ligand-driven shifts in ACh potency have previously been identified for ligands interacting with nAChRs allosterically, as performed with PNU-120596 and desformylflustrabromine (66, 77). Another clue that SCoV2P is an allosteric modulator can be observed in the SCoV2P concentration response data (Fig. 2). At the peak potentiating concentration (10 μM) of SCoV2P, the enhancement in potentiated response for the nicotine 200 nM and 300 nM treatment groups matches well with the percentage of receptors desensitized (Fig. 2*D*). As there is no increase in receptor pool due to nicotine incubation (Fig. 3*B*), these data show that with potentiating concentrations, SCoV2P can transition nicotine desensitized α7 nAChRs back to the resting state (Fig. 10*C*). We also observed a significant reduction in ACh potency with a high, inhibiting concentration of SCoV2P. These findings suggest that SCoV2P may bind with different orientations and likely occupies an allosteric site on α7 nAChRs to perform its dual functional actions.

Similar allosteric actions of SCoV2P were not observed on heteromeric nAChRs. At high concentrations, SCoV2P inhibited ACh-induced currents of α4β2, α3β2, and α3β4 nAChRs, with some isoform specific effects (Fig. 1). In the absence of nicotine, the α4β2, α3β2, and α3β4 subtypes were minimally inhibited. With pretreatment and cotreatment of 200 nM nicotine, SCoV2P inhibition of (α4β2)_2_α4 and (α3β2)_2_α3 ACh-mediated currents were enhanced. The inhibition of (α3β2)_2_α3 ACh-mediated currents reached similar levels to the α7 nAChR (∼40% inhibition of ACh-induced currents). These results, in combination with the α7 nAChR data, suggest that α subunits, and perhaps α/α interfaces, may contain the SCoV2P-binding site. These findings demonstrate that nAChR subtypes interact with the SARS-CoV-2 neurotoxin-like region, which may have cellular consequences, such as mediating viral entry.

While preparing this article, Chrestia *et al*. (2022) published a nice body of work identifying that the neurotoxin-like region of SARS-CoV-2 (Y674-R685) acts as a silent-agonist to cause α7 nAChR currents when applied with PNU-120596 and inhibits α7 nAChRs by a proposed noncompetitive mechanism (53). To facilitate a more direct comparison to our work, we evaluated the functional outcomes of a shortened peptide containing the last 16 residues of SCoV2P (Y674-S689). We identified that Y674-S689 has similar positive allosteric modulation activities to that of SCoV2P, as it needs an agonist to have its effects (Fig. 6). One potential difference that may explain the macroscopic observational differences between the presented work and Chresita *et al*. (2022) is that we used a slightly longer peptide. We chose this as these residues were in SCoV2P and are found in the ectodomain. Another difference is the type of TEVC system. We use a relatively fast perfusion system where the oocyte is flushed with fresh drugs or buffer solutions at a rate of 4 ml/min, while Chresita *et al*. (2022) used a system where the oocyte is dunked into 230 μl of stationary solution and thus the drug to buffer exchange is slower (53). Importantly, both bodies of work contribute to our knowledge regarding how the SARS-CoV-2 glycoprotein interacts and modulates α7 nAChRs. Chrestia *et al*. (2022) single channel unitary studies identify that the low concentrations of the neurotoxin-like region enhance α7 nAChR transition to the active conformation in the presence of ACh or PNU 120596 (53). Godellas *et al*. (2022) also released a key paper while the presented work was under review which showed that the SARS-CoV-2 spike protein, specifically regions S375-L390 and Y674-R685, do not compete with ACh, choline or nicotine for binding to the orthosteric site on α7 nAChRs, which is consistent with our conclusions that the mechanism of action is *via* an allosteric site (54).

The work presented in this article demonstrates at low concentrations SCoV2P and SCoV2ED positively modulate ACh-mediated currents *via* facilitation of α7 nAChRs transition to the active conformation. The modulation activity switches to inhibition at higher concentrations of both SCoV2P and SCoV2ED. Further, we provide visual confirmation that SCoV2P interacts with α7 nAChRs transfected in neuronal-like N2a and HEK293 cultured cells (Figs. 7 and 8). Cell culture imaging results demonstrate that SCoV2P interacts preferentially to the surface of cells expressing α7 nAChRs, which is consistent with our functional data. Non-transfected HEK293 cells, which do not endogenously express nAChRs, showed little residual fluorescence. These findings demonstrate that α7 nAChRs are cellular targets of the SCoV2P.

COVID-19 disease severity has been linked to an over-production of pro-inflammatory cytokines promoting what is referred to as a cytokine storm (78). α7 nAChRs are found prevalently in the immune system in a multitude of cell types including macrophages, T cells, dendritic cells, and B-cells (39, 79, 80). Activation of α7 nAChRs in the cholinergic anti-inflammatory pathway suppresses the production of pro-inflammatory cytokines (81, 82, 83). Cells in the immune system express nAChR subunits including α3, α4, β2, and β3 (84, 85, 86) and applying the noncompetitive antagonist mecamylamine has been shown to reduce the pro-inflammatory response (87).

As α7 nAChR potentiation suppresses the immune system (83), it is tempting to speculate that α7 nAChR positive modulation by the SARS-CoV-2 neurotoxin-like region may allow for rapid and minimally restricted viral replication early in COVID-19 infection due to activation of the cholinergic anti-inflammatory response. In support of this theory, the delta SCoV2P enhanced ACh potentiation in two ways: an increase in total potentiation and a decrease in the concentration required to induce ACh potentiation. The potency for the inhibition phase was also enhanced. Together these factors may provide some explanation as to why patients infected with the SARS-CoV-2 delta variant experience reduced incubation time, worse symptoms, and increased fatality (88, 89, 90). These potential scenarios may be amplified in smokers, as these individuals have enhanced expression of nAChRs, many of which are desensitized. With the ability of SCoV2ED to potentiate α7 nAChRs (Fig. 9) and, in further detail, the SARS-CoV-2 neurotoxin-like region to resensitize nicotine desensitized α7 nAChRs (Fig. 2), tobacco users’ cholinergic anti-inflammatory response may be further activated, thus allowing for even higher levels of viral replication to occur. Our data also suggests that with nicotine, the effects of SCoV2P become broader to include α4β2 and α3β2 subtypes in addition to α7 nAChRs. The increased disease severity and mortality rate for smokers may be due to these proposed modulatory effects of the SARS-CoV-2 on the immune system. Additional investigations probing the above speculation could shed light onto disease pathophysiology.

Nicotine pretreatment enhances SCoV2P and SCoV2ED modulation of α7 nAChR responses resulting in enhanced potentiation *via* a mechanism that resensitizes nicotine desensitized receptors. This conclusion has potentially large epidemiological consequences as there are 1.3 billion tobacco users worldwide according to the World Health Organization. This raises the concern that physiological processes modulated by α7 nAChRs, such as the immune system and the cholinergic anti-inflammatory response, may be further compromised in tobacco users who are infected with SARS-CoV-2. Understanding how the SARS-CoV-2 glycoprotein and the neurotoxin-like region affects different nAChR subtypes and associated isoforms provides an enriched understanding of COVID-19 pathophysiology and will likely facilitate the development of targeted therapeutics.

## Experimental procedures

### Reagents

Acetylcholine chloride, atropine sulfate, bovine serum albumin (BSA), (−)-nicotine, and other reagents were purchased from Sigma Aldrich. SCoV2P, FITC-SCoV2P, delta-SCoV2P, and Y674-S689 were designed by M.M. Weltzin and purchased from Elim BioPharmaceuticals with HPLC purity >90%. The unmutated SCoV2ED was purchased from Thermo Fisher Scientific. Fresh solution stocks were made daily and diluted as required.

### DNA constructs and cRNA synthesis

Human α3 (NM_000743.5), α4 (NM_000744.5), α7 (NM_000746.3), β2 (NM_000748.2), and β4 (NM_000750.5) nAChR subunit complementary DNAs were generously gifted from Drs Ron J. Lukas and Paul Whiteaker in mammalian expression vectors (pCI [Promega]) for all constructs, except the α7 subtype, which is in pSHE, a modified pGEMHE vector. The DNA plasmid for the chaperone protein NACHO in pREP9 was generously gifted by Dr R. Loring (Northeastern University). α7 pSHE DNA was transferred into pcDNA 3.1(+) by inserting the gene first into pCI using the restriction enzymes XbaI and NotI then into pcDNA3.1(+) using XbaI and XhoI. The α7-pHuji construct was purchased from Invitrogen GeneArt Gene Synthesis Services (Thermo Fisher Scientific) in the pMA-RQ vector and moved into the mammalian expression vector pcDNA3.1(+). DNA constructs were digested with their respective enzymes and separated on a 0.8% ethidium bromide agarose gel. Appropriate vector and insert bands were cut out and purified using the Wizard SV Gel and PCR Clean-up system. All cDNAs were amplified for use by transformations using NEB 5-α competent *Escherichia. coli* cells (New England Biolabs [NEB]). DNA isolation was accomplished using Qiagen QIAprep Spin Miniprep kits. Extracted DNA was verified by restriction enzyme digest (XbaI and NotI for constructs in the pCI vector and XbaI and EcoRV for constructs in pSHE vector and XbaI, NotI and PvuI for pcDNA3.1 (+)) (NEB) and visualized on a 1% ethidium bromide agarose gel. To achieve transfection-grade constructs for cell culture experiments, α7, α7-pHuji, and NACHO plasmid DNA was amplified using the EndoFree Plasmid Maxi Kit.

In preparation for cRNA synthesis, cDNA subunits in the pCI vector were linearized with the restriction enzyme SwaI, while the α7-pSHE construct used NheI. Linearized DNAs were visualized by gel electrophoresis, treated with proteinase K (NEB) to digest any contaminating proteins, and purified utilizing the Qiagen PCR clean-up kit. Linearized templates were then used to transcribe cRNA using the T7 mMESSAGE mMACHINE High Yield Capped RNA Transcription Kit (Ambion). Confirmation of cRNA purity was accomplished *via* quantification using the NanoDrop 2000 and electrophoresis gel imaging. Final products were stored at −80 °C in subaliquots.

### Oocyte preparation and cRNA injection

Stage IV and V *X. laevis* oocytes were purchased from IACUC-certified Ecocyte Bioscience for human nAChR expression. All efforts were made to minimize animal suffering and to reduce the number of animals used. The α7 homomeric nAChR was expressed in oocytes by injection of the individual subunit. For co-expression of α7 nAChRs with NACHO, a 40 ng α7 subunit to 0.4 ng NACHO cRNA injection ratio was used. To express the desired heteromeric nAChR subtype, cRNAs were injected into oocytes in biased ratios to enhance specific isoform expression. High agonist sensitivity nAChRs received a higher percentage of the β subunit cRNA than the α subunit. The low agonist sensitivity nAChRs were expressed by injecting higher percentage of the α subunit to the β subunit cRNA into oocytes, as outlined in Table S5. Each oocyte received a cRNA injection of 81 nl *via* impalement using a pulled micropipette with an outer diameter of ∼40 μm. Oocytes were incubated in buffer (82.5 mM NaCl, 2.5 mM KCl, 1 mM MgCl_2_, 1 mM CaCl_2_, 1 mM Na_2_HPO_4_, 5 mM Hepes, 600 μM theophylline, 2.5 mM sodium pyruvate, 50 μg/ml each penicillin, streptomycin, neomycin, gentamycin sulfate, pH to 7.5 using NaOH) at 13 °C for 72 to 120 h prior to recording, with daily buffer changes.

### Electrophysiology

TEVC electrophysiology was used to measure nAChR function. No less than 72h post-cRNA injection, *X. laevis* oocytes expressing α7, α4β2, α3β2, or α3β4 nAChRs were voltage clamped at −70 mV using an Axoclamp 900A amplifier (Molecular Devices, LLC). Data acquisition and analysis were performed using pClamp 10.6 software (Molecular Devices; https://www.moleculardevices.com/). Direct current offset was accomplished using a 40 Hz high-pass filter and 10 kHz low-pass Bessel filter. Recording electrodes were pulled from thin wall capillary glass and filled with 3 M KCl. Electrode resistance ranged from 0.5 to 10 MΩ. Oocytes with leak currents below −100 nA were discarded.

Drug solutions were applied to clamped oocytes using a 16 channel, gravity-fed, perfusion system with automated valve control (AutoMate Scientific, Inc). All drug solutions were made in an oocyte ringer 2 (OR_2_) recording buffer (92.5 mM NaCl, 2.5 mM KCl, 1 mM MgCl_2_·6H_2_O, 1 mM CaCl_2_·2H_2_O, 5 mM Hepes, pH to 7.5 using NaOH) containing atropine sulfate (1.5 μM) and 0.1% BSA. Atropine sulfate was used to block potential response from endogenous muscarinic receptors, and BSA was used to prevent SCoV2P and SCoV2ED from sticking to plastics in the experimental apparatus. Solutions were made fresh daily. The SCoV2ED came prepared in a PBS 7.2 pH buffer, which we diluted to 40% in our OR_2_ recording buffer to minimize buffer effects.

nAChR ACh pharmacology is dependent on the individual subunits that assemble to form distinct subtypes and heteromeric receptor isoforms (91, 92). ACh concentration-response profiles were generated to ensure the correct nAChR subtype and isoform expression. Potency of ACh was determined by applying increasing concentrations of ACh (0.010 μM - 10 mM) for 1 s with an 84 s wash of OR_2_ recording buffer in between each drug application (Fig. S4). Using the generated ACh peak current responses, we determined the α7 nAChR ACh potency to be 183 μM, which matches literature values (Fig. S4*A* and Table S6) (33).

Heteromeric nAChRs express in at least two isoforms, each with unique sensitivities to ACh (33, 93, 94, 95). Considering the α4β2 subtype, the monophasically-fit (α4β2)_2_β2 isoform has high sensitivity to ACh (Fig. S4*B* and Table S6). The (α4β2)_2_α4 isoform ACh concentration-response profile was best fit with a biphasic curve with a high ACh sensitivity component and a second lower sensitivity phase (Fig. S4*B* and Table S6). The ACh response profiles for the α3β2 isoforms were both monophasic with high ((α3β2)_2_β2) and low potencies ((α3β2)_2_α4) (Fig. S4*C* and Table S6). Within the α3β4 subtype, the (α3β4)_2_β4 isoform displayed high ACh potency, while the (α3β4)_2_α3 isoform had lower ACh sensitivity (Fig. S4*D* and Table S6). The measured ACh potencies for all the tested nAChR subtypes and isoforms were comparable to literature values (33, 93, 94, 95). Using our cRNA subunit injection preparations, we successfully expressed the α7 subtype and isoforms of the α4β2, α3β2, and α3β4 nAChRs.

To determine the effects of the SCoV2P on the tested nAChR subtypes, increasing concentrations of peptide (0.01–300 μM) were preapplied for 30 s followed by 1 s of ACh at the subtype-specific EC_90_ concentration, followed by 225 s of OR_2_ recording buffer (Table S6). Responses were normalized to the ACh EC_90_ prior to the SCoV2P application. Peptide concentration-response profiles were also performed on α7 nAChRs for delta SCoV2P and control peptide RVG-P, as described above (Fig. 5*A*). For experiments involving nicotine, nAChR-expressing oocytes were preincubated with 100, 200, or 300 nM nicotine for 80 to 110 min. Nicotine was present in all drug and buffer solutions.

To determine the SCoV2P modulation mechanism, α7 nAChR ACh concentration-response profiles were conducted in the presence of 10 or 100 μM SCoV2P. For each drug application, SCoV2P was preapplied for 30 s followed by 1 s of ACh at increasing concentrations (1 μM–10 mM), followed by 89 s of OR_2_ recording buffer. Responses were normalized to the ACh EC_100_ without peptide preapplication. For experiments involving nicotine, nAChR-expressing oocytes were preincubated for 80 to 110 min with 200 nM nicotine. During these experiments, nicotine was present in all drug and buffer solutions. To establish if nicotine chaperoned α7 nAChRs during our preincubation period, ACh-evoked currents were measured on either naïve or nicotine treated oocytes. To determine the length of time it took to wash off nicotine, 1 s ACh EC_90_ currents were measured, followed by 4 min buffer washes. Recovery was determined when successive ACh applications produced equitable responses (difference < 2%).

ACh concentration-response profiles were also performed for 1 μM Y674-S689. Using our standard protocol, 1 μM Y674-S689 was preapplied for 30 s followed by 1 s of ACh at increasing concentrations (1 μM–10 mM), followed by 89 s of OR_2_ recording buffer. Responses were normalized to the ACh EC_100_ without peptide preapplication. These experiments were also performed for oocytes co-expressing α7 nAChRs and NACHO (Fig. 6).

In order to further elucidate SCoV2P’s modulation mechanism, several other experiments were performed. Experiments were performed in gap-free mode with manual valve operation. To verify that SCoV2P effects were mediated by α7 nAChRs, 10 nM MLA was coapplied with 3 μM SCoV2P for 30 s followed by a 2 s ACh EC_90_ application. To evaluate whether Y674-S689 exhibited the silent agonism seen by Chrestia *et al* (53), 1 μM Y674-S689 was coapplied for 30 s with either 3 μM or 10 μM PNU-120596. To demonstrate if SCoV2P shares a common binding site with PNU-120596 or is a silent agonist, 3 μM PNU-120596 was coapplied with 3 μM SCoV2P for 30 s. The resulting modulation of α7 nAChRs was evaluated using an ACh EC_90_-evoked response (2 s). To determine if SCoV2P could enhance steady-state responses, α7 nAChRs were exposed to either 60 μM (EC_20_) or 30 mM (EC_100_) for ∼30 s until steady-state was achieved. In attempts to evoke a response during steady-state, 3 μM SCoV2P was coapplied with ACh. Reactivation of nicotine desensitized receptors experiments were accomplished by preincubating α7-nAChR expressing oocytes as described, followed by a 30 s application of 3 μM SCoV2P and a 2 s ACh EC_90_ stimulation.

For evaluation of SCoV2ED effects on α7 nAChRs, each oocyte was placed in incubation buffer with 40% PBS and a single concentration of the ectodomain for 5 min. Oocytes were then rapidly voltage clamped and the α7 nAChRs were acutely stimulated with a 1 s application of ACh (EC_90_). At each SCoV2ED concentration (0.01–30 nM), 25 to 44 oocytes were used. Responses were normalized to a control group exposed to incubation buffer with 40% PBS without SCoV2ED. Another control group not exposed to PBS was included demonstrating differences in recording buffer composition.

To further explore the potentiation seen at 0.1 nM SCoV2ED, ACh concentration-response profiles were performed. For each drug application, SCoV2ED was preapplied for 60 s followed by 1 s of ACh at increasing concentrations (1 μM–10 mM), followed by 89 s of OR_2_ recording buffer. Responses were normalized to the ACh EC_100_ without SCoV2ED preapplication. Evaluation of the effects of the SCoV2ED on 200 nM nicotine desensitized α7 nAChRs, oocytes were exposed to 200 nM nicotine for 80 to 110 min, followed by a preincubation of 0.1 nM SCoV2ED with 200 nM nicotine present. The resulting effect on α7 nAChRs was measured at either the ACh EC_20_ or EC_90_.

### Cell culture and transient transfection

Mouse neuroblastoma N2a cells and HEK293 cells were purchased from American Tissue Culture Collection and cultured in Eagle’s Minimum Essential Medium with 10% fetal bovine serum and 1× penicillin-streptomycin (VWR). Cultures were maintained at 37 °C and 5% CO_2_ in a humidified cell culture incubator. Cells were subcultured onto poly-D-lysine-coated glass coverslips (MatTek) and transiently transfected when approximately 70% confluent, using the Lipofectamine 2000 transfection reagent (Invitrogen) for N2a cells or Calcium Phosphate Transfection Kit (Millipore Sigma) for HEK293 cells. Mammalian α7 nAChR or α7-pHuji nAChR coding plasmid DNA and NACHO chaperone plasmid DNA were used at a 4:1 ratio following the manufacturers’ protocols. Transfected cells were incubated for 24 h (N2a) or 48 h (HEK293) before addition of peptide.

### Fluorescent peptide imaging

To assess interactions with N2a or HEK293 cells, 50 μM FITC-tagged SCoV2P was added to the growth medium 24 h or 48 h post transfection and incubated for an additional 24 h. Cells were extensively rinsed with pH 7.4 PBS three times before being imaged on the Olympus Fluoview FV10i Laser Scanning Confocal Microscope and processed using ImageJ (https://imagej.nih.gov/ij/download.html). For imaging of FITC tags, the excitation wavelength was 495 nm, while the emission wavelength was 519 nm. Cells transfected with α7-pHuji were also imaged at 566 nm excitation wavelength and 598-nm emission wavelength. FITC and pHuji bleed-through were not observed at either emission wavelength. For colocalization imaging, images were taken at the cell surface level to show surface interactions. ImageJ was used for postprocessing of images (NIH) (94), including deconvolution using the DeconvolutionLab2 and PSF Generator plug-ins (both Biomedical Imaging Group, EPFL).

To determine CTCF with ImageJ, we calculated the mean integrated density of fluorescence of our cell groups, using 2D images, by free-hand tracing the outlines of cells of interest in the phase channel, to reduce operator bias. Area, mean, and integrated density were measured in the channel of the fluorescent tag. While viewing only with the phase channel on, visually healthy cells were selected. Three separate regions of interest were traced surrounding each selected cell to account for background fluorescence. Using these values, CTCF was calculated using the following formula: CTCF = integrated density – (area of cell x mean background fluorescence) (http://theolb.readthedocs.io/en/latest/imaging/measuring-cell-fluorescence-using-imagej.html#measuring-cell-fluorescence-using-imagej). This process was repeated for 30 cells for each group.

### Cell viability assay

N2a cells were plated on 48-well plates (VWR) and α7 nAChR-positive groups were transfected as described above. Twenty-four hours post transfection, cells were exposed to varying concentrations of SCoV2P and incubated for another 24 h. As positive control for cell death, 15% DMSO was added to control wells instead of peptide. Media was changed post incubation and alamarBlue cell viability (Thermo Fisher Scientific) reagent was added to all wells according to the manufacturer’s suggestions. Cells were incubated for 3 h before fluorescent measurements were collected on a Tecan Spark Multimode Microplate Reader (Tecan U.S., Inc).

### Data analysis

All TEVC experiments were conducted on at least two oocyte isolations and two batches of cRNA synthesis. Throughout the article, large N indicates the number of replicates and small n indicates the number of individual oocytes. All data analysis was accomplished using GraphPad Prism 9.3 software (www.graphpad.com). TEVC EC_50_, IC_50_, Hill slopes (n_H_), and ACh EC_20_ and EC_90_ current amplitude (I_EC20_ or I_EC90_) values were determined from individual oocytes. All generated curves were calculated using standard built in nonlinear curve fitting. A sum of squares F-test was used to determine whether an unconstrained monophasic sigmoidal or a constrained biphasic logistic equation best fit the ACh concentration-response data. The inhibitory phase of the SCoV2P concentration-response profiles were fit to monophasic nonlinear curves. SCoV2ED inhibition data was fit with a monophasic nonlinear regression curve with a constrained hill slop (n_H_ = −1). Statistical analyses were performed using GraphPad Prism 9.3. Data was analyzed using Welch’s two tailed *t* test to compare pairs of groups. One-way ANOVA with Tukey’s or Dunnett’s multiple comparison test were used to evaluate the means of three or more groups.

## Data availability

All data are available in the main text or the supplementary materials.

## Supporting information

This article contains supporting information.

## Conflict of interest

The authors declare that they have no conflicts of interest with the contents of this article.
