# Supplementary material for: SARS-CoV-2 spike ectodomain targets α7 nicotinic acetylcholine receptors
Source: J Biol Chem. 2023 Apr 13;299(5):104707. doi: 10.1016/j.jbc.2023.104707 (PMC10101490; doi:10.1016/j.jbc.2023.104707)
Supplement: Supporting information [file mmc1.docx]

Supporting Information for

**SARS-CoV-2 spike ectodomain targets α7 nicotinic acetylcholine receptors**

Brittany C. V. O’Brien^1^, Lahra Weber^1^, Karsten Hueffer^2^ and Maegan M. Weltzin^1^*

*Correspondence to: mmweltzin@alaska.edu

**This PDF file includes:**

Figs. S1 to S6

Tables S1 to S6


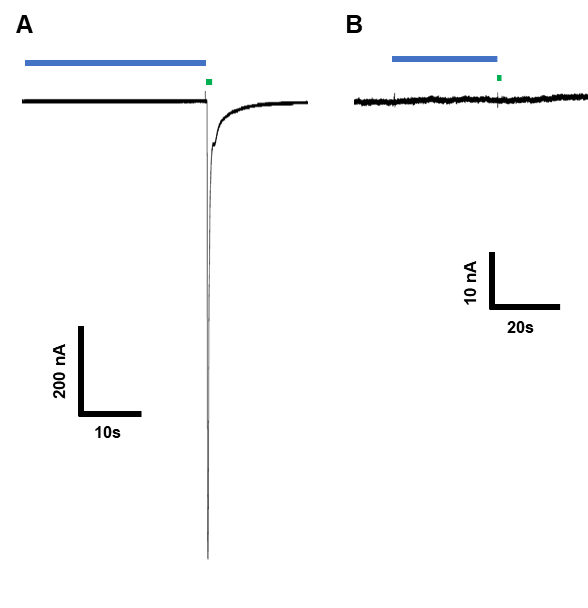


**Fig. S1. SCoV2P cannot directly activate nAChRs. (A)** α7-nAChR had no visible response to application of 10 μM SCoV2P (blue). Ability to respond to stimulus was confirmed by application of 1300 μM ACh (green). **(B)** Un-injected oocytes were unresponsive to 10 μM SCoV2P or 1300 μM ACh. All experiments were performed in triplicate.


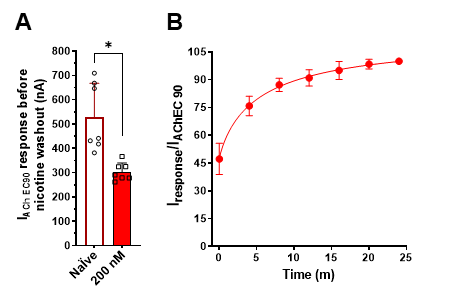


**Fig. S2. α7-nAChR recovery from nicotine desensitization. (A)** Pre-exposure of 200 nM nicotine for 80-100min desensitized ~50% of the total receptor pool. This data set was collected using the first ACh application in order to track the full recovery profile (Welch’s two-tailed t-test, *t*(3.4)=4.08, *P=0.0209). **(B)** Nicotine-desensitized receptors are capable of recovery when no longer exposed to nicotine. Recovery of the full activatable receptor pool takes approximately 24min. Data are mean±S.D (N=3, n=7-9).

**
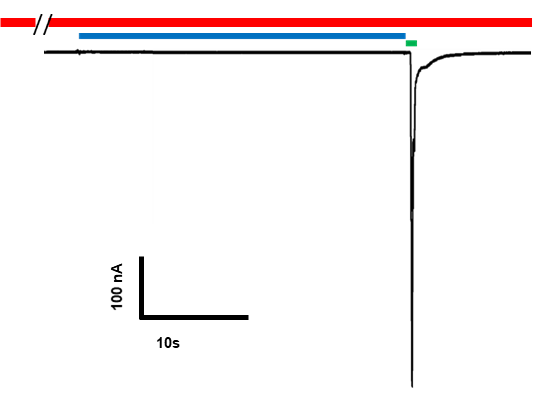
**

**Fig. S3. SCoV2P cannot directly reactivate nicotine desensitized α7 nAChRs**. After 80-110min of sub-activating exposure to 200 nM nicotine (red), 3 μM SCoV2P (blue) was applied to determine if SCoV2P could transition receptors directly from the desensitized to the open state. No activation was seen with 30s of peptide application but α7 nAChRs were responsive to a 1s ACh (green) EC_90_ stimulation. Experiment was performed in triplicate.

**
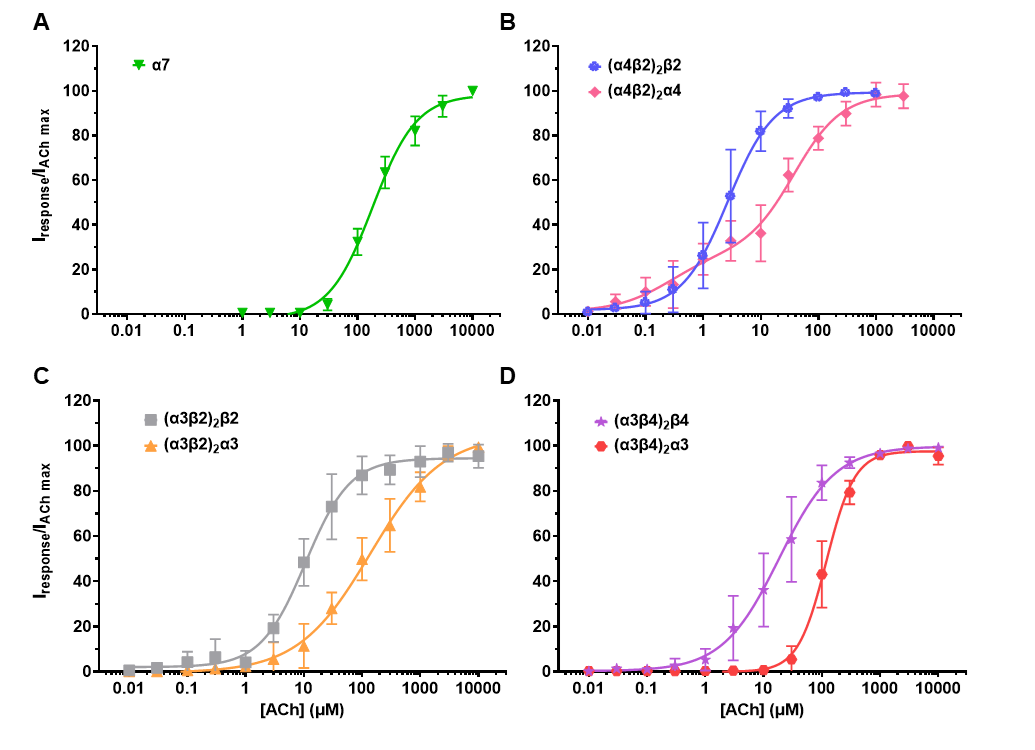
**

**Fig. S4. Acetylcholine concentration-response profiles for α7 and isoforms of α4β2, α3β2 and α3β4 nAChR.** Fitted parameter values can be found in Table S6. Calculated potency (EC_50_) values are consistent with the literature, demonstrating correct isoform expression. Data points are mean±S.D.

**Fig. S5. Modulation of ACh-induced current is sequence specific.** Normalized RVG-P induced alterations in ACh-mediated responses as a percentage of control response. Previously presented SCoV2P data is included to facilitate comparison. RVG-P, which contains high sequence homology in the neurotoxin-like domain of SCoV2P, does not produce a potentiation effect, and has an inhibition potency approximately 30-fold higher than that of SCoV2P. Data points are mean±S.D (RVG-P N=5, n=7).

**
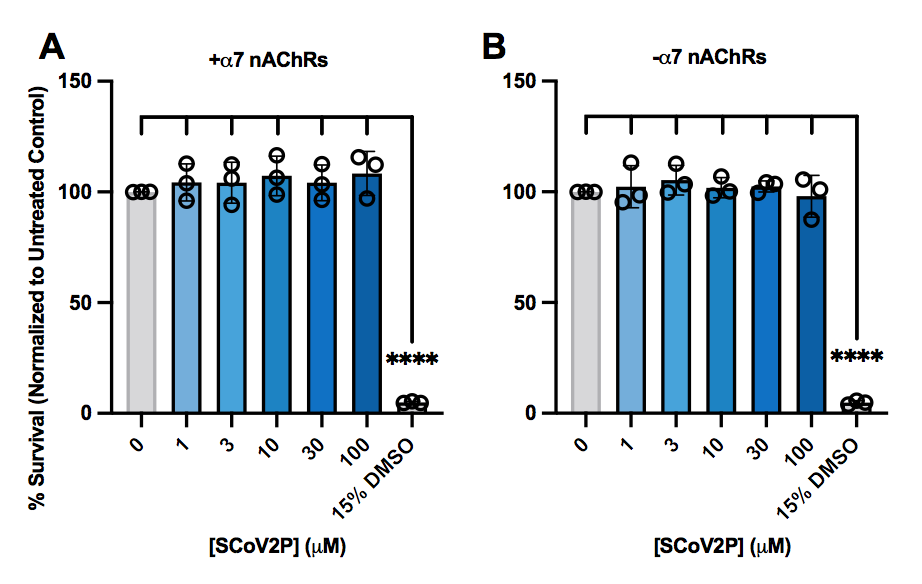
**

**Fig. S6. SCoV2P is not cytotoxic.** SCoV2P did not show significant cytotoxic effects on N2a cells transiently transfected with α7 and NACHO plasmid DNA **(A)** or non-transfected N2a cells **(B)** at concentrations ranging from 1-100 μM SCoV2P (N=3, n=3) (One-Way ANOVA with Tuckey’s multiple comparisons test, ^ns^P=0.8-1.0, ****P<0.0001). Cells were exposed to SCoV2P or 15% DMSO for 24h before conducting an alamarBlue cell viability assay. All points are the mean±SD.

**Table S1. Sequence comparison between the SARS-CoV-2 spike glycoprotein (alpha (B.1.1.7)) and other regions of viral glycoproteins with neurotoxin-like regions similar to α-bungarotoxin.** The neurotoxin-like regions in α-bungarotoxin and the rabies glycoprotein interact with nAChRs. Bolded residues are those conserved between two or more proteins. RVG-P has the identical sequence as the dog rabies virus glycoprotein in the region of interest, except for an M amino acid mutation at T187.

| **Virus/Toxin** |  | **Sequence** | | | | | | | | | | | | | | | | | | | | | | | | | | | | | | |
| --- | --- | --- | --- | --- | --- | --- | --- | --- | --- | --- | --- | --- | --- | --- | --- | --- | --- | --- | --- | --- | --- | --- | --- | --- | --- | --- | --- | --- | --- | --- | --- | --- |
| α-Bungarotoxin | **Y** | R | K | M | **W** | - | - | - | - | - | - | - | - | - | - | **C** | **D** | A | **F** | C | **S** | **S** | **-** | **R** | **G** | **K** | **V** | V | E | L | **G** | 40 |
| SARS-CoV-2 Spike | **Y** | - | E | C | D | I | **P** | I | **G** | A | G | I | C | A | **S** | Y | Q | T | Q | **T** | **N** | **S** | P | **R** | R | A | **R** | S | V | A | S | 689 |
| Dog Rabies virus | **Y** | T | - | I | **W** | **M** | **P** | E | N | P | R | L | G | T | **S** | **C** | **D** | **I** | **F** | **T** | **N** | **S** | **-** | **R** | **G** | **K** | **R** | A | S | K | **G** | 203 |
| HIV gp120 | S | G | G | M | I | **M** | E | K | **G** | E | I | K | N | C | **S** | F | N | **I** | S | **T** | **S** | I | **-** | **R** | **G** | **K** | **V** | Q | K | E | Y | 173 |

**Table S2. Data for SCoV2P concentration-response profiles in Fig. 2B.** Inhibition data was fit with an antagonist monophasic non-linear regression curve. See Fig. 2B for additional statistics.

| **α7 nAChR Nicotine Treatment** | **N (n)** | **[0.01 *μ*M] SCoV2P Response ± SD (%)** | **Peak Potentiation ± SD (%)** | **IC_50_ *(μM)***  **[95% CI]** |
| --- | --- | --- | --- | --- |
| None | 5 (8) | 102 ± 5 | 111 ± 8^†3^ | 339  [273, 460] |
| 100 nM Nicotine | 3 (7) | 106 ± 2 | 117 ± 7 | 247  [214, 286] |
| 200 nM Nicotine | 5 (7) | 112 ± 5 | 131 ± 7**^1A^ | 240  [204, 280] |
| 300 nM Nicotine | 3 (9) | 121 ± 4 | 135 ± 10**^1B^ | 197*^2^  [166, 231] |

Footnotes

^1^Significance calculated as difference from untreated α7 nAChR by One-way ANOVA with Tukey’s multiple comparison test F(2,10)=16.95, (A) **P=0.0016 and (B) **P=0.0015.

^2^Significance calculated as difference from untreated α7 nAChR by One-way ANOVA with Tukey’s multiple comparison test F(3,12)=4.54, *P=0.0246.

^3^Significance calculated as difference from baseline 100% response by one sample t-test, t=3.406, df=4, ^†^P=0.0271.

**Table S3. Calculated parameters for SCoV2P ACh concentration-response profiles.** Data was fit with monophasic curves as determined by a sum of squares F-test using GraphPad Prism 9.1. See Fig. 4B for additional statistics.

| **α7 nAChR Treatment** | **N (n)** | **EC_50_ (*μM)***  **[95% CI]** | **n_H_**  **[95% CI]** | **Peak Response ± SD (%)** |
| --- | --- | --- | --- | --- |
| None | 6 (14) | 192  [174, 212] | 1.2  [1.1, 1.4] | 99.9 ± 0.3 |
| 10 μM SCoV2P | 4 (9) | 119**^1A^  [104, 138] | 1.2  [1.1, 1.5] | 144 ± 8****^2A^ |
| 200 nM Nicotine + 10 μM SCoV2P | 5 (11) | 121**^1B^  [103, 150] | 1.1  [1.0, 1.4] | 163 ± 18****^2B^ |
| 100 μM SCoV2P | 3 (7) | 287  [195, 481] | 0.8  [0.6, 1.0] | 73 ± 9***^2C^ |

Footnotes

^1^Significance calculated as difference from untreated α7 nAChR by One-way ANOVA with Tukey’s multiple comparison test F(2,12)=13.80, (A) **P=.0018 and (B) **P=0.0014.

^2^Significance calculated as difference from untreated α7 nAChR by One-way ANOVA with Tukey’s multiple comparison test F(3,13) = 144.8, (A and B) ****P<0.0001, (C) ***P=0.0003.

**Table S4. Calculated parameters for Y674-S689 ACh concentration-response profiles.** Data was fit with monophasic curves as determined by a sum of squares F-test using GraphPad Prism 9.1. See Fig. 6 for additional statistics.

| **α7 nAChR Treatment** | **N (n)** | **EC_50_ (*μM)***  **[95% CI]** | **n_H_**  **[95% CI]** | **Peak Response ± SD (%)** |
| --- | --- | --- | --- | --- |
| None | 6 (14) | 192  [174, 212] | 1.2  [1.1, 1.4] | 99.9 ± 0.3 |
| 1 µM Y674-S689 | 4 (14) | 93****^1^  [85, 102] | 1.8  [1.6, 2.1] | 127 ± 11*^4^ |
| NACHO | 3 (6) | 188^ns 3^  [172, 206] | 1.2  [1.1, 1.3] | 99 ± 1 |
| NACHO +  1 µM Y674-S689 | 3 (6) | 70****^2, ns 3^  [58, 85] | 1.6  [1.3, 1.9] | 118 ± 10 |

Footnotes

^1^Significance calculated as difference from untreated α7 nAChR by One-way ANOVA with Tukey’s multiple comparison test F(3, 9) = 69.12, ****P<0.0001.

^2^Significance calculated as difference from NACHO α7 nAChR by One-way ANOVA with Tukey’s multiple comparison test F(3, 9) = 69.12, ****P<0.0001.

^3^Significance calculated as difference from matching treatment without NACHO by One-way ANOVA with Tukey’s multiple comparison test F(3, 9) = 69.12, ^ns^P>0.05.

^4^Significance calculated as difference from untreated α7 nAChR by One-way ANOVA with Tukey’s multiple comparison test F(4, 11) = 4.876, *P=0.037.

**Table S5. cRNA injection ratio details used to express isoforms for each nAChR subtype.** Injected cRNA ratios were optimized and verified using ACh concentration-response profiles. The accessory subunit is in bold type.

| **nAChR Subtype and Isoform** | **α : β subunit cRNA Ratio** | **Total cRNA Injected (ng)** |
| --- | --- | --- |
| α7 | N/A | 40.0 |
| (α4β2)_2_**β2** | 1:10 α4:β2 | 11.0 |
| (α4β2)_2_**α4** | 100:1 α4:β2 | 12.6 |
| (α3β2)_2_**β2** | 1:30 α3:β2 | 31.0 |
| (α3β2)_2_**α3** | 30:1 α3:β2 | 31.0 |
| (α3β4)_2_**β4** | 1:30 α3:β4 | 31.0 |
| (α3β4)_2_**α3** | 30:1 α3:β4 | 31.0 |

**Table S6. Calculated parameters for ACh concentration-response profiles shown in Fig. S4.** Data was fit with either a biphasic or monophasic curve as determined by a sum of squares F-test in GraphPad Prism 9.1. As anticipated, only (α4β2)_2_α4 data fit biphasically.

| **nAChR**  **Subtype** | **N (n)** | ***EC_50 1_* *(μM)***  **[95% CI]** | **n_H 1_**  **[95% CI]** | ***EC_50 2_* *(μM)***  **[95% CI]** | **n_H 2_**  **[95% CI]** | ***EC_90_* *(μM)***  **[95% CI]** |
| --- | --- | --- | --- | --- | --- | --- |
| α7 | 6 (14) | 192  [174, 212] | 1.2  [1.1, 1.4] | N/A | N/A | 1250  [975, 1650] |
| ***α4β2*** | | | | | | |
| (α4β2)_2_**β2** | 5 (14) | 2.6  [2.2, 3.0] | 1.0  [0.9, 1.2] | N/A | N/A | 21.4  [14.9, 31.9] |
| (α4β2)_2_**α4** | 6 (13) | 0.2  [0.06, 4.9] | 0.8  [0.4, 1.5] | 38.8  [26.6, 59.6] | 0.98  [0.81, 1.74] | 374  [206, 963] |
| ***α3β2*** | | | | | | |
| (α3β2)_2_**β2** | 5 (14) | 10.0  [8.6, 11.6] | 1.1  [0.9, 1.2] | N/A | N/A | 79.4  [57.9, 112] |
| (α3β2)_2_**α3** | 4 (9) | 139  [110, 181] | 0.7  [0.6, 0.8] | N/A | N/A | 3050  [1820, 5560] |
| ***α3β4*** | | | | | | |
| (α3β4)_2_**β4** | 6 (15) | 18.2  [15.6, 21.2] | 0.9  [0.8, 1.0] | N/A | N/A | 216  [158, 302] |
| (α3β4)_2_**α3** | 5 (12) | 119  [111, 128] | 1.8  [1.6, 2.0] | N/A | N/A | 409  [348, 484] |
